# Novel Ultrasound-Responsive Amyloid Formulation

**DOI:** 10.3390/ph17060777

**Published:** 2024-06-13

**Authors:** Maytham Ismail, Mathumai Kanapathipillai

**Affiliations:** Department of Mechanical Engineering, University of Michigan-Dearborn, Dearborn, MI 48128, USA; mgismail@umich.edu

**Keywords:** amyloid aggregates, thermoresponsive polymer, drug delivery, RIP3

## Abstract

Amyloid aggregates have attracted significant interest in regard to diverse biomedical applications, particularly in the field of drug delivery. Here, we report novel amyloid aggregates based on a 12-amino-acid peptide from the amyloidogenic region of the receptor-interacting kinase 3 (RIP3) protein and a thermoresponsive triblock copolymer, namely, Pluronic F127 (RIP3/F127). Physicochemical characterization was performed to determine the aggregation size, morphology, and stimuli-responsive properties. The potential of the aggregates as a drug depot was assessed in lung cancer cells, using Doxorubicin (Dox) as a model drug. The results show that RIP3 and RIP3/F127 exhibit amyloidogenic properties. Further, the RIP3/F127 amyloids exhibited significant ultrasound-responsive properties compared to amyloid aggregates without Pluronic F127. Moreover, the RIP3/F127/Dox amyloid formulations that were subjected to ultrasound treatment exhibited greater toxicity to lung cancer cells compared to that of Dox alone at equal concentrations. Overall, the results from this proof-of-concept study show that amyloidogenic peptide aggregates with stimuli-responsive properties can be utilized as efficient drug delivery depots.

## 1. Introduction

Protein aggregation can have adverse or beneficial effects on cellular functions [1]. Conventionally, they are linked to pathological forms that are hallmarks of many aging diseases. Recently, there has been interest in investigating and utilizing beneficial forms of protein and/or peptide amyloid aggregates that are prevalent in physiological systems [2]. One such protein family is the receptor-interacting protein kinase (RIP) family. Among the RIP proteins, it has been widely shown that RIP1/RIP3 kinase proteins form amyloid aggregates to facilitate necroptosis [3]. Several studies have reported on the amyloid formation and characterization of RIP1/RIP3 proteins. Recently, short amino acid sequences of the RIP1/RIP3 proteins have also been shown to form amyloid-like structures [3,4,5]. In addition to its role in necroptosis, RIP3 overexpression has been shown to have inhibitory effects in some cancers [6,7]. Hence, utilizing the amyloid structures of the RIP3 protein and/or peptide could have beneficial effects in cancer therapeutic applications.

Stimuli-responsive drug delivery strategies have an additional advantage over conventional drug delivery: they can be tuned to deliver a drug in a spatial, temporal, and controlled-release manner. Several stimuli-responsive drug depots have been developed, including those responsive to temperature, ultrasound, magnetic field, light, and pH [8]. Among the various stimuli, ultrasound has attractive features due to its noninvasive, patient-friendly delivery; deep tissue penetration; thermal and mechanical modulation; and direct cytosolic drug delivery [9,10]. Microbubbles, polymeric nanoparticles, and recently echogenic exosomes have been explored in regard to ultrasound-mediated delivery [11,12,13]. Ultrasound-mediated drug delivery utilizing biomaterials has been used in several therapeutic applications, including cancer treatment [14,15]. However, the use of amyloid aggregates alongside ultrasound stimuli has not been widely explored when compared to research on conventional nanocarriers. Due to their intrinsic therapeutic properties, physiologically beneficial amyloids could have significant therapeutic efficacy and hence a significant impact in drug delivery applications.

Pluronic F127 is a thermoresponsive polymer with a lower critical solution temperature (LCST) property [16,17]. Pluronic F127 is an FDA-approved polymer and has been utilized as a versatile biomaterial in nanomedicine [18,19]. Due to its non-toxic and biocompatible properties, it has been used in various biomedical applications, including drug delivery [20]. Several studies have reported that Pluronic F127-based biomaterials can be tuned to impart several stimuli-responsive properties. Moreover, Pluronic polymers have been shown to impart both thermo- and mechanosensitive properties to materials. Studies reveal that Pluronic micelles alone or in combination with polymeric materials induce drug release in the presence of ultrasound stimuli [21,22]. The application of ultrasound leads to a temporary increase in temperature, which then drives the LCST-based phase change in Pluronic F127, leading to increased drug release [22]. Pluronic F127 has also been used as a combination therapy in various composite drug delivery systems [19,23]. Like Pluronic-F127-incorporated polymeric systems, the incorporation of Pluronic-F127 in amyloid-based structures can enhance the stimuli-responsive properties of amyloids and be utilized in drug delivery applications.

In this study, we investigated whether peptide/polymer-based amyloid aggregates could be used as drug depots. The amyloidogenic 12-amino-acid NIYNCSGVQVGD sequence of the RIP3 proteins together with the thermoresponsive Pluronic F127 polymer, used to impart stimuli-responsive properties, were used to form the amyloid structures. This is the first time amyloid-like structures composed of Pluronic F127 have been investigated. The physicochemical characterization and ultrasound-mediated stimuli-responsive properties of the amyloids were studied. In addition, cellular uptake and cellular efficacy studies were performed. The potential of the amyloid aggregates as a drug delivery depot was tested with Doxorubicin (Dox) as a model drug, and the efficacy of the amyloid Doxorubicin formulation was tested in A549 lung cancer cells. The study reported herein could serve as a platform for peptide/polymer-based amyloid structures in stimuli-responsive drug delivery.

## 2. Results

**Figure 1:**
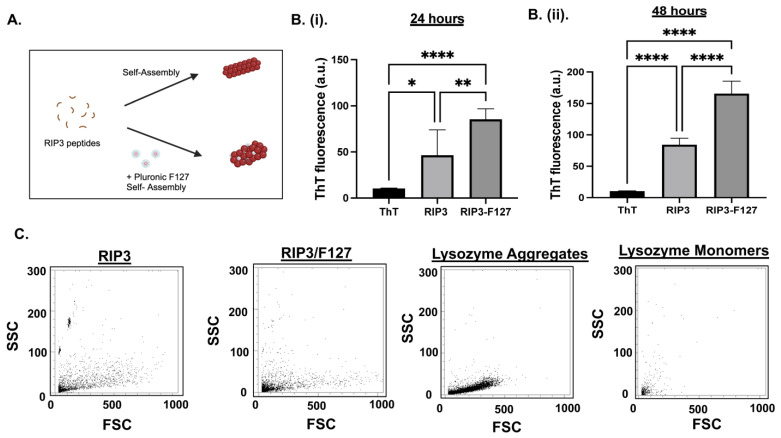
(**A**) Schematic of RIP3 amyloid aggregation formation with and without Pluronic F127. (**B**) Aggregation of RIP3 peptides with and without F127 characterization was investigated using ThT fluorescence. Measurements were carried out at 24 h (**i**) and 48 h (**ii**) after the initiation of aggregation. At least three independent experiments were carried out. * *p* < 0.05, ** *p* < 0.01, and **** *p* < 0.0001. (**C**) Flow cytometry analysis of Proteostat dye assay of RIP3 and/or RIP3/F127 aggregation. Lysozyme aggregates and monomers were used for comparison. Samples aggregated for 48 h were used for the assay.

First, the aggregation kinetics of 200 µM of RIP3 peptides with and without Pluronic F127 0.01% were studied. A schematic of the aggregation formation process is depicted in Figure 1A. ThT fluorescence measurements were taken to measure the amyloid aggregation formation. The results show that both the RIP3 and/or RIP3/F127 formulations exhibited significant ThT fluorescence (Figure 1B). Further, the samples containing Pluronic F127 exhibited significant aggregation compared to the RIP3 peptide samples alone, as evidenced by increased ThT fluorescence (Figure 1B). In addition, an increase in ThT fluorescence at 48 h compared to 24 h was observed (Figure 1B(i,ii)). Aggregation formation was also confirmed using a flow-cytometry-based Proteostat assay. Lysozyme protein aggregates and monomers were used as standards for comparison. Both RIP3 and RIP3/F127 exhibited protein aggregate properties like scattering (Figure 1C). Furthermore, RIP3 showed complex structures, as evidenced by significant side scattering compared to RIP3/F127 and/or lysozyme protein aggregates. In addition, the final content ratio of RIP3/F127 in the amyloid aggregates was quantified. RIP3-Rhodamine and FITC-F127 were used for the amyloid formation. Fluorescence measurements revealed that 39.9 ± 2.51% and 90.43 ± 3.28% of the initial amounts of peptide and F127 used for the amyloid aggregation were retained in the final formulation.

**Figure 2:**
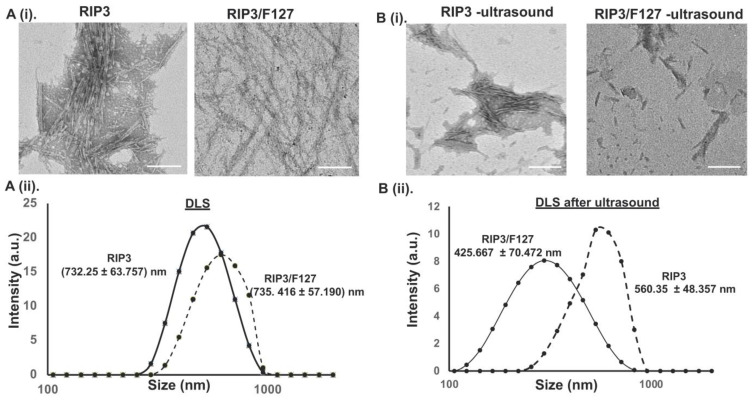
(**A**) (**i**) The morphologies of amyloid structures were characterized using TEM. Scale bar: 200 nm. (**ii**) DLS measurements of RIP3 amyloids with and without Pluronic F127. (**B**) (**i**) TEM images of the amyloid aggregates before and after subjection to ultrasound stimuli (1.6 W/m^2^, for 3 min). Scale bar: 200 nm. (**ii**) DLS measurements of the amyloid aggregates before and after they were subjected to ultrasound.

Next, TEM was used to characterize the morphology and size of the aggregates. After 48 h of aggregation, RIP3 peptides with and without Pluronic F127 exhibited an amyloid-like morphology. The RIP3 peptide samples revealed a more compact fibrous-like structure compared to the RIP3/F127 samples, which showed less dense fibrillar structures (Figure 2A(i)). Both amyloid structures exhibited fiber lengths in the range of 500–1000 nm. In addition to TEM, DLS measurements were used to determine the size of the amyloid structures. The average sizes were found to be 732.25 ± 63.757 nm and 735.416 ± 57.190 nm for the RIP3 and RIP3/F127 samples, respectively (Figure 2A(ii)).

To determine whether the amyloid aggregates impart any stimuli-responsive properties, their mechanosensitive characteristics were investigated. The effect of ultrasound stimuli (1.6 W/cm^2^ for 3 min) on the amyloid aggregate structures was examined using TEM and DLS measurements. As can be seen from the TEM images in Figure 2B(i), the amyloid structures were broken down into smaller fibers compared to the amyloid structures that were not subjected to any ultrasound stimuli, suggesting ultrasound-responsive properties. Further, RIP3 amyloid structures with F127 showed greater ultrasound responsiveness than the RIP3 aggregates alone. In addition, the size of the aggregates before and after ultrasound was measured using DLS (Figure 2B(ii)) to corroborate the findings. The average size of the RIP3 aggregates subjected to ultrasound was reduced to 560.35 ± 48.357 nm, and the average size of RIP3/F127 aggregates was reduced to 425.667 ± 70.472 nm.

**Figure 3:**
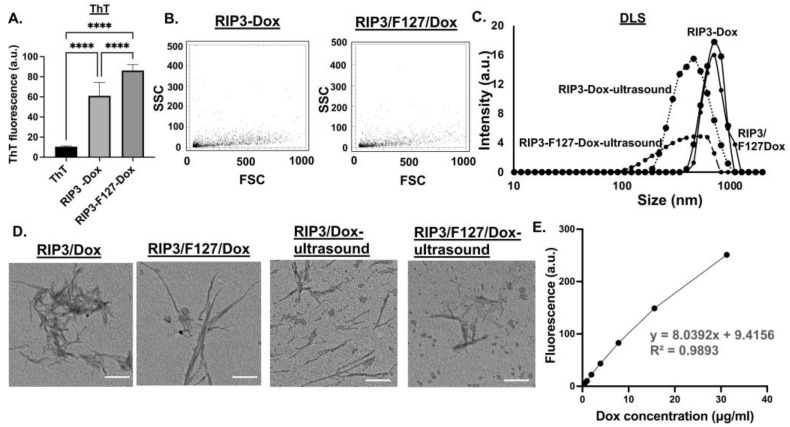
Amyloids with Dox were characterized using (**A**) ThT and (**B**) Proteostat assay. Three independent experiments were carried out. **** *p* < 0.0001. Ultrasound measurements (1.6 W/m^2^ for 3 min) were taken for the amyloid–Dox formulations. DLS measurements (**C**) and TEM images (scale bar 200 nm) (**D**) were obtained before and after ultrasound application. Three independent measurements were taken. (**E**) Dox encapsulation was determined from the dox calibration curve obtained at 470/495 nm excitation/emission. Dox concentrations of 0.5–50 µg/mL were used. Three independent experiments were used to determine encapsulation efficiency.

To investigate the potential of the aggregates as drug depots, the amyloid aggregates were formulated with and without Dox, and their physicochemical properties were characterized after 48 h. First, ThT measurements were taken to confirm aggregation. As can be seen from Figure 3A, both RIP3/Dox and/or RIP3/F127/Dox amyloid formulations showed significant ThT fluorescence compared to ThT alone, indicating aggregation. Further, as shown by the reduced ThT fluorescence, the incorporation of Dox seems to have reduced the aggregation compared to RIP3 peptides alone. As a complementary technique, Proteostat measurements were taken. Aggregate properties like light scattering was observed for both the RIP3/Dox and RIP3/F127/Dox samples, indicating the formation of aggregates in the presence of Dox (Figure 3B). DLS and TEM images were also obtained to further characterize the amyloid aggregates (Figure 3). The results indicate a reduction in aggregation compared to that for the samples without Dox, as shown by ThT, TEM, and DLS measurements (Figure 3A–D). Next, we tested whether the aggregates impart ultrasound-responsive properties. As can be seen from the TEM and DLS measurements, ultrasound stimulation resulted in smaller amyloid structures and sizes (*p*-values < 0.05), indicating responsive properties (Figure 3C,D). The DLS measurements reveal RIP3/Dox aggregate sizes of 657.514 ± 57.403 nm and 462.386 ± 57.930 nm before and after ultrasound stimulation (*p*-value < 0.05), and for RIP3/F127/Dox, aggregate sizes of 716.467 ± 124.115 nm and 333.283 ± 59.583 nm were observed (*p*-value < 0.01). Moreover, compared to RIP3/F127, the RIP3/F127/Dox aggregates exhibited significant responsive properties. Table 1 summarizes the size measurements.

Since RIP3/F127/Dox showed significant ultrasound-responsive properties, it was used for further investigation in cellular efficacy studies. To calculate the amount of Dox encapsulated in the RIP3/F127/Dox–amyloid aggregates, calibration curves were created (Figure 3E). Dox concentrations of 0.5–50 µg/mL were used to generate the fluorescence curve at 470/595 nm excitation/emission. The amount of Dox was then calculated from the curve (y = 8.0392x + 9.4156). It was found that the encapsulation efficiency was 49.523 ± 3.821%. Next, Dox release measurements were taken at 24 h and 48 h. The percentages of Dox released were calculated based on the Dox calibration curve and found to be 56.168 ± 1.915 and 81.231 ± 0.907%. The results are summarized in Table 2.

**Figure 4:**
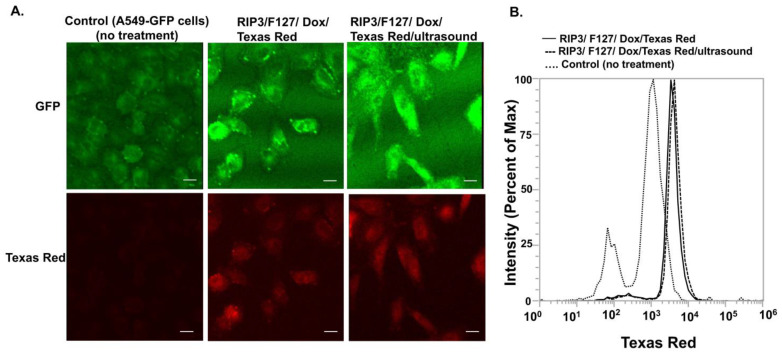
The cellular uptake of the amyloid aggregates was investigated using confocal microscopy and flow cytometry. A549 cells were treated with amyloid aggregates for 30 min, and then uptake was analyzed. (**A**). The cellular uptake of the amyloid aggregates was investigated using confocal microscopy. Scale bar 20 µm. (**B**). Flow cytometry measurements depict the shift in fluorescence with and without aggregate treatment.

**Figure 5:**
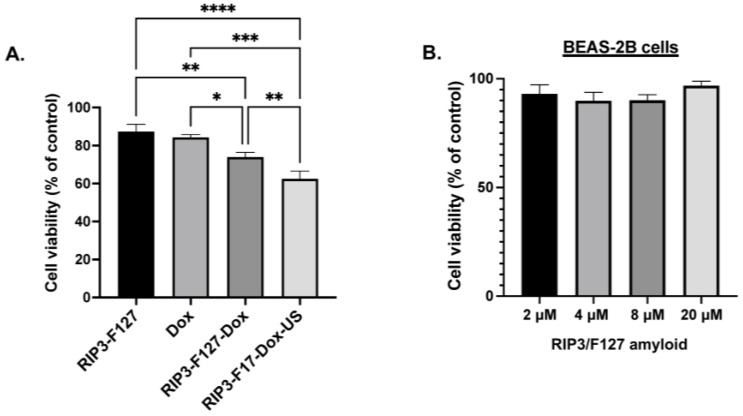
(**A**). Effects of the amyloid drug depots on A549 lung cancer cells were assessed using alamarBlue measurements. A549 lung cancer cells were plated in 96 wells at a density of 10,000 cells/well and treated with free Dox, amyloid aggregates with and without Dox, and/or ultrasound (US) at a concentration of 2.5 µM. Three independent experiments were performed. * *p* < 0.05, ** *p* < 0.01, *** *p* < 0.001, and **** *p* < 0.0001. (**B**). Effects of the amyloid drug depots on BEAS-2B normal lung epithelial cells. BEAS-2B lung epithelial cells were plated in 96 wells at a density of 10,000 cells/well and treated with amyloid aggregates, and the metabolic activity was analyzed with alamarBlue after 48 h.

Finally, we investigated the potential of RIP3/F127/Dox amyloids for use in lung cancer treatment. A549 GFP lung cancer cells were used for cellular efficacy studies. First, cellular uptake of the aggregates was confirmed using confocal microscopy and/or flow cytometry. Cells were treated with Texas-Red-labeled amyloid aggregates composed of RIP3/F127/Dox and/or RIP3/F127/Dox subjected to ultrasound stimuli (1.6 W/m^2^, 3 min) for 30 min, and confocal imaging and flow cytometry measurements were carried out. As can be seen from Figure 4A, the cells treated with Dox–amyloid aggregates showed enhanced Texas Red fluorescence with and without ultrasound application, indicating cellular uptake. The findings were next corroborated with the results of the flow cytometry studies. As shown in Figure 4B, a small increase in the uptake for the ultrasound-stimulated samples compared to the non-ultrasound-treated samples was observed from the shift in the Texas Red fluorescence. Next, cells were treated with RIP3/F127 peptide aggregates with and without Dox and/or with and without ultrasound, and toxicity measurements were carried out after 48 h. The results show that the Dox-encapsulated aggregates induced greater toxicity than free Dox (Figure 5A). In addition, the ultrasound-treated Dox aggregates exhibited greater toxicity compared to the Dox aggregates that were not subjected to ultrasound treatment. Moreover, amyloid aggregates (RIP3/F127) did not exhibit toxicity to normal lung epithelial cells at concentrations in the range of 2 to 20 µM, as shown in Figure 5B.

## 3. Discussion

In this manuscript, we have investigated the potential of ultrasound-responsive peptide/polymer amyloidogenic aggregates as drug depots. A short amino acid sequence from the amyloidogenic-prone regions of the RIP3 protein has been shown to form amyloid-like aggregates. Further, the physicochemical characteristics of the RIP3 aggregates were tuned via the incorporation of the thermoresponsive polymer Pluronic F127, which, when incorporated with Dox, exhibited more significant anticancer properties compared to the Dox-free aggregates. The toxic effects were further enhanced with ultrasound-stimulated amyloid formulations. This is the first time an amyloid structure composed of an amyloidogenic peptide and a Pluronic polymer has been investigated. The self-assembling properties of Pluronic existing because of its micellar structures may have facilitated the self-assembly with the RIP3 peptide resulting in peptide/polymer amyloid fibers. Further, the RIP3/F127 amyloid structures exhibited significant ultrasound-mediated stimuli-responsive properties as evidenced by DLS, TEM, and flow cytometry. This may be due to the solvating effects of Pluronic F127 within the amyloid structure resulting in loosely bonded amyloid fibers. The incorporation of the hydrophilic chemotherapeutic drug Dox in the amyloid structures could have occurred either via self-assembly or via the interaction of the Dox molecules with the amino acids of the RIP3 peptide and/or Pluronic F127.

The results from this proof-of-concept study show the promise of peptide/polymer-based amyloid aggregate formulations. In addition, we recently reported the successful amyloid formulation of RIP3/RIP1-protein-based short peptides and their potential in cisplatin drug delivery [4]. However, to realize the full potential of these amyloid-based structures as drug depots, future studies need to focus on further optimization and conducting additional investigations. The amyloid structures reported in this study are quite large, and hence formulation conditions need to be tuned to achieve smaller amyloids with a narrow distribution. Also due to the fiber-like structures, DLS measurements may not reflect the actual size of amyloids, and hence complementary size measurements such as those yielded by atomic microscopy and or scanning electron microscopy would strengthen the characterization and understanding of the structures. Further, the mechanism of drug incorporation and the resulting amyloid structures need to be thoroughly investigated. Additionally, although the results show the successful uptake of the amyloids by the cells, the exact mechanism of this uptake needs to be investigated. Studies on pathologic abeta aggregates have reported macropinocytosis to be the major endocytosis mechanism of cellular uptake [24,25]. Each amyloid structure has its own distinct characteristics, and hence a detailed cellular uptake mechanism of RIP3-based structures should be studied in future investigations.

Finally, there has been a lot of interest recently in utilizing the amyloidogenic properties of proteins and peptides in therapeutic applications [2,26,27,28]. The work presented here supports the notion that amyloid-based nanomaterials could be used in therapeutic applications, such as drug delivery. Further, the properties of amyloids could be tuned by combining them with stimuli-responsive polymers. Pluronic F127 polymers are well-studied thermoresponsive polymers that have been utilized in drug delivery and tissue engineering applications [23,29,30]. In addition to their thermoresponsive properties, these polymers can be tuned to impart pH- and mechanosensitive properties, hence the added advantage. Hence, the combination of amyloidogenic peptides with a stimuli-responsive polymer such as Pluronic F127 could lead to the development of novel biomaterial platforms that could be utilized in various biomedical applications.

## 4. Materials and Methods

### 4.1. Materials

Receptor-interacting protein RIP 3 peptide NIYNCSGVQVGD was custom-synthesized from Genscript (Piscataway, NJ, USA). Thioflavin-T (ThT) (T3516), Pluronic F127 (P2443), DMSO (276855), Ammonium acetate (A1542), Trizma hydrochloride (T15760), Phosphotungstic acid (HT152), and Doxorubicin hydrochloride (44583) were obtained from Millipore Sigma (Milwaukee, WI, USA). PROTEOSTAT protein aggregation assay (ENZ-510233) were obtained from Enzo Life Sciences (Farmingdale, NY, USA). Texas Red (T6134) and NHS-Rhodamine (46406) were obtained from Thermo Fisher Scientific (Waltham, MA, USA), and FITC-PEG-NH_2_ was obtained from Nanocs (New York, NY, USA). The cell culture reagents RPMI medium, fetal bovine serum, and antibiotic-antimycotic were obtained from Thermo Fisher Scientific (Waltham, MA, USA).

### 4.2. Amyloid Formulation

For the amyloid formulation, peptide containing the 12-amino-acid sequence NIYNCSGVQVGD from the aggregation-prone region of the RIP3 protein was used. First, a 50 mM RIP3 peptide stock solution was prepared in DMSO. Next, 200 µM peptide solutions were obtained by diluting the peptide in 100 mM ammonium acetate buffer (pH 7), and amyloid formation was initiated at 37 °C in a thermomixer. In addition, peptide solutions were mixed with and without Pluronic F127 (0.01% *w*/*v*) and or Dox (50–100 µg/mL), and amyloid formation was investigated. Amyloids encapsulated with Texas Red were used for imaging studies. To remove free peptides, Dox, and/or Texas Red, aggregates were purified via centrifugal filtration (MWCO 3400 Da). To determine the final peptide and Pluronic F127 content in the amyloid aggregates, first, RIP3 peptides were labeled with Rhodamine-NHS, and Pluronic F127 was labeled with FITC-PEG-NH_2_. The labeled peptide and Pluronic F127 were then used to form the amyloid aggregates in 100 mM ammonium acetate buffer (pH 7), and after 48 h, the aggregates were purified via centrifugal filtration (MWCO 3400 Da). Next, the amounts of RIP3 and F127 were determined using fluorescence measurements of Rhodamine (552/575 nm) and FITC (498/517 nm) excitation/emission.

### 4.3. Thioflavin T (ThT) Fluorescence and Proteostat Assay

Aggregation was confirmed using ThT and Proteostat assay. A total of 100 µL of 50 µM of ThT in 20 mM of Trizma hydrochloride (Tris) buffer, pH 8, was mixed with 5 µL of the amyloid sample, and the aggregation formation was assessed at 24 h and 48 h at 440/482 nm excitation and emission. A SpectraMax M3 spectrophotometer from Molecular Devices was used for the measurements, and at least three independent experiments were performed. In addition to ThT, Proteostat assay (Enzo Life Sciences) was used. This method is widely used for monitoring peptide aggregation [4,31]. Samples were prepared as per the manufacturer’s protocol, and aggregation was compared with lysozyme protein monomer and aggregate standards using flow cytometry.

### 4.4. Size and Morphology

Dynamic Light-Scattering (DLS) measurement was used to determine the size of the amyloids. First, samples were diluted ten times. A total of 50 µL of distilled water was mixed with 5 µL of the amyloid sample in a cuvette, and size measurements were carried out using a Malvern zeta sizer instrument. To assess morphology, Transmission Electron Microscopy (TEM) imaging was performed. Samples were placed on a 200-mesh copper grid and stained with 2% phosphotungstic acid at pH 7.4. Imaging was carried out utilizing a JEOL TEM microscope at the University of Michigan Ann Arbor Medical School’s Electron Microscopy Facility.

### 4.5. Ultrasound Stimuli Measurements

To assess whether the aggregates had mechanical responsive properties, ultrasound stimuli experiments were performed. For this study, ultrasound was applied by using an ultrasonicator 740 from Mettler Electronic Corp. (Anaheim, CA, intensity 1.6 W/cm^2^) for three minutes. The effect of ultrasonication on the aggregates was measured via DLS, TEM, and flow cytometry.

### 4.6. Doxorubicin (Dox) Encapsulation and Release Study

Dox encapsulation and release studies were performed to determine the dox concentration for the efficacy studies. First, a dox fluorescence vs. concentration standard calibration curve was obtained. Dox concentrations in the range of 0.5–50 µg/mL were used, and the fluorescence measurement was performed at 470/595 nm excitation and emission, as reported elsewhere [32,33,34]. Next, the amount of dox in the amyloid formulation was calculated based on the standard calibration curve (linear equation: y = 8.0392x + 9.4156). Encapsulation efficiency was determined according to the ratio of the Dox amount used in the formulation at the start of the aggregation to that of the amount in the formulation after purification via centrifugal filtration (MWCO 3 kDa). To determine the degree of Dox release, aggregated samples were incubated at 37 °C in ammonium acetate buffer, and the amount of Dox released at 24 and 48 h was calculated using the standard curve. The Dox release percentage was determined according to the ratio of dox released at the measured time point to that of the total Dox at time zero.

### 4.7. Cellular Efficacy Studies

For the cellular toxicity study, A549 GFP cells, which have been widely used as a model for investigating lung cancer, were used. The cells were regularly cultured in the lab. Cells were grown in RPMI, 10% fetal bovine serum, and 1% antibiotic-antimycotic. Cells were cultured at 10,000 cells/well in a 96-well plate. After 24 h, the cells were treated with 2.5, 5, or 10 μM of Dox alone or 2 to 20 µM of RIP3/F127 amyloid aggregates or RIP3/F127 amyloid aggregates encapsulated with 2.5 or 5 or 10 μM of Dox before and/or after ultrasound (1.6 W/cm^2^, for 3 min). After 48 h of incubation, alamarBlue measurements were taken. Cell toxicity was assessed according to the metabolic activity of the cells at 570/590 nm excitation/emission. Toxicity studies were also carried out in BEAS-2B normal lung epithelial cells to test whether the aggregates induced any toxicity in non-tumorigenic cells [35,36]. Cells were cultured in RPMI supplemented with 10% fetal bovine serum and 1% antibiotic-antimycotic. Next, cells were plated in a 96 well-plate at a density of 10,000 cells/well. After 24 h, cells were treated with RIP3/F127 amyloid aggregates at concentrations ranging from 2 to 20 µM. Cells were then incubated for 48 h and subsequently stained with alamarBlue, and the metabolic activity was assessed at 570/590 nm excitation/emission.

In addition, the cellular uptake of the amyloid aggregates was investigated using confocal imaging and flow cytometry. The aggregates were made using Texas Red for the flow cytometry and confocal studies. For the flow cytometry analysis, first, cells were cultured at 50,000 cells/well in a 24 well plate. After 24 h, the RIP3/F127/Dox amyloid aggregates before and/or after being subjected to ultrasound stimulation (1.6 W/cm^2^, for 3 min) were added to the cells for 30 min. Next, cells were trypsinized, resuspended in PBS buffer, and kept on ice. The uptake was assessed using flow cytometry within 30 min of trypsinization using an Attune flow cytometer in the lab. For the flow cytometry test, flow conditions were set at 10, 000 events (cell number), and 488 nm blue excitation laser with emission filters of 530/30 nm and 574/26 nm was used to monitor GFP-labelled A549 cells and Texas-Red-labelled aggregates, respectively. Additionally, confocal imaging was performed to confirm cellular uptake of the amyloids. For confocal imaging, a similar cell culture procedure was used. Briefly, cells were cultured at 50,000 cells/well in a 24-well plate at a density of 50,000 cells/well, and after 24 h, RIP3/F127/Dox amyloid aggregates before and/or after being subjected to ultrasound stimulation (1.6 W/cm^2^, for 3 min) were added to the cells for 30 min. Cells were then washed three times with sterile PBS buffer. Next, cells were fixed with 4% PFA for 20 min and subsequently washed three times with PBS. Cells were then imaged with a Leica confocal microscope in the lab. Multiple images were taken for each treatment condition.

### 4.8. Statistical Analysis

Each experiment was performed at least three times. The average ± standard error of the mean (SEM) was used to describe the data. The statistical significance was determined using the one-way analysis of variance (ANOVA), followed by Tukey’s HSD post hoc analysis. The significance of the data is denoted by the following symbols: ns—nonsignificant, * *p* < 0.05, ** *p* < 0.01, *** *p* <0.001, and **** *p* < 0.0001.

## Figures and Tables

**Table 1:** Summary of DLS size measurements.

Aggregates	Size (nm)
RIP3-Dox	657.514 ± 57.403
RIP3-Dox-ultrasound	462.386 ± 57.930
RIP3/F127/Dox	716.467 ± 124.115
RIP3/F127/Dox-ultrasound	333.283 ± 59.583

**Table 2:** Summary of Doxorubicin encapsulation and release.

Doxorubicin (Dox) Amyloid Formulation	Encapsulation Efficiency (%)	Drug Release (%)	
		24 h	48 h
RIP3/Dox/F127	39.926 ± 6.308	56.168 ± 1.915	81.231 ± 0.907

## Data Availability

The data supporting the results of the article will be made available by the authors, without any restrictions.
